# Implications of Mobile Technology on Hospitalization Rates in Medically Underserved Areas Worldwide: A Systematic Review

**DOI:** 10.7759/cureus.78409

**Published:** 2025-02-03

**Authors:** Matthew Heffernan, Rahul Mittal, Barbara Tafuto

**Affiliations:** 1 Health Informatics, Rutgers University, New Brunswick, USA

**Keywords:** smart health wearable, wearable biosensing devices, wearable devices, wearable electronic devices, wearable health devices

## Abstract

Hospitalizations in medically underserved areas remain a significant challenge, often driven by barriers such as limited access to healthcare services due to institutional barriers or physical facility access due to distance and transportation issues, inadequate preventive care, and a higher prevalence of chronic diseases. In recent years, mobile health (mHealth) technology has emerged as a promising tool to bridge healthcare gaps, offering innovative solutions to address these challenges by leveraging mobile devices to improve patient care, health monitoring, and engagement. However, while digital and mHealth interventions hold promise, implementing them in underserved areas presents unique challenges. This scoping review aims to assess the current landscape of mobile technology interventions in medically underserved areas, with a particular focus on their effects on hospitalization rates.

A systematic review was conducted utilizing the PubMed, Embase, and CINAHL databases for the identification of articles to include within the review. After using the search terms in each database, a total of 416 articles were found to meet the search query. All articles were screened and reviewed based on the criteria, and 15 articles (n=15) were selected for review.

The most commonly studied technologies were telehealth visits (n=9), while mobile apps (n=4), smart glasses (n=1), and remote patient monitoring devices (n=1) made up the remaining technologies studied. Hospitalizations were grouped as either inpatient hospitalizations (n=8), emergency department (ED) visits (n=4), or other hospital referrals in cases where a patient may be referred to a hospital but was not classified as either inpatient hospitalization or emergency. Across the emergency group studies, the experimental arm of the studies had a lower rate of ED visits and inpatient admissions.

There could exist potential complications for evaluating these new digital technologies in rural and underserved populations, including level of education and cost. Additionally, while 15 articles were reviewed in this systematic review, there appears to be substantially more literature available for non-rural, non-underserved communities. Within the limited data currently available, we found reductions in inpatient admission rates and ED visits when utilizing digital technologies. Given the potential cost benefits that these technologies could provide, further investigation into this topic may be warranted.

## Introduction and background

Terminology

Medically Underserved Area

Some geographic areas experience a shortage of healthcare services, resulting in limited access to essential medical care for residents.

Mobile Health

The utilization of mobile devices to support the provision of healthcare involves using smartphones, tablets, and other portable technology to enhance access to medical services, improve communication between healthcare providers and patients, and enable remote monitoring and consultations.

Telehealth

The provision of healthcare remotely through telecommunications technology in discussion with healthcare providers.

Introduction

The processes that begin with patient diagnosis and culminate inpatient treatment remain a significant challenge in medically underserved areas worldwide, especially inpatient care in a hospital setting. These challenges are often driven or exacerbated by barriers such as limited access to healthcare services, inadequate preventive care, and a higher prevalence of chronic diseases, such as chronic obstructive pulmonary disease (COPD), coronary heart disease, diabetes, or stroke [1]. Medically underserved populations, particularly those in rural and low-income regions, face unique healthcare disparities that contribute to higher rates of mortality, preventable hospital admissions, and poorer health outcomes [2]. In recent years, mobile health (mHealth) technology has emerged as a promising tool to bridge healthcare gaps, offering innovative solutions to address these challenges by leveraging mobile devices to improve patient care, health monitoring, and engagement [3].

Mobile technology, including applications for telemedicine, remote monitoring, and health education, provides a flexible and accessible approach to managing health outside of traditional clinical settings. These tools have the potential to reduce hospitalizations by promoting preventive care, enhancing chronic disease management, and empowering patients to take an active role in their health [4]. However, while digital and mHealth interventions hold promise, implementing them in underserved areas presents unique challenges, such as limited digital literacy, inconsistent access to technology, and variable user engagement, with disparities including uneven availability to access telemedicine visits and reduced utilization among minority patients in the United States [5]. Understanding the impact, barriers, and outcomes of mHealth initiatives in these communities is essential to evaluating their effectiveness and scalability.

The worldwide lockdowns from the SARS-COV-2 pandemic have reshaped healthcare delivery, pushing traditionally in-person models of care to new, digital alternatives, including increasing the proportion of hospitals offering telemedicine and remote monitoring services from 47% in 2017 to 72% in 2021, with numbers of visits increasing from 111.4 million telemedicine visits in 2020 to 194.4 million visits in 2021 in the United States, and demonstrating promise in assisting patients with self-management and outcomes [4-7].

This scoping review aims to assess the current landscape of mobile technology interventions in medically underserved areas worldwide, including indigenous and rural regions within their home countries. It focuses particularly on their effects on the frequency of acute care or emergency department (ED) visits, inpatient hospital admissions, or other hospital referrals, especially in cases where hospital visit or referral rates were discussed but, due to differences in local healthcare structures, the categorization of hospital visits was irregular. These are hereafter referred to as "hospitalizations." By synthesizing available evidence, this review seeks to identify how mHealth tools can support healthcare in underserved communities, highlight gaps in the existing literature, and provide recommendations for future research and policy development. Through this comprehensive overview, we aim to contribute insights into the role of mobile technology in reducing hospitalizations and improving overall health outcomes, such as improved health and reduced cost of care, in vulnerable populations.

## Review

Methods

A systematic review was conducted utilizing the PubMed, Embase, and CINAHL databases for the identification of articles to include within the review. The search strategy included keywords and MeSH terms related to wearable technology, digital health technology, hospitalization rates, and medically underserved areas. Boolean operators were used to combine terms as follows: "Rural population OR area, medically underserved OR underserved population, medically OR physician shortage area OR area, physician shortage" AND "Biomedical technology OR digital technology OR wearable electronic devices OR fitness trackers OR Smart glasses OR mobile applications OR wearable computer OR wearable technology OR wearable biosensing devices OR smart health wearable OR wearable device for gait rehabilitation OR wearable health devices OR wearable sleep technology OR wearable wristbands" AND "Hospitalization OR patient admission OR emergency service, medical OR emergency medical services OR emergency room visits OR emergency service, hospital OR admission, patient OR admissions, patient."

Bibliographies of identified relevant studies were also manually reviewed to identify any additional relevant articles. Studies were included in the review if they investigated the use of wearable or digital health devices, reported either the total number of hospitalizations or hospitalization rate as an outcome measure, focused on patients residing in medically underserved areas, involved human subjects, and were published as a peer-reviewed article in the English language. Only articles written between 2009 and 2024 were considered for inclusion to ensure the most up-to-date literature was considered.

Studies were excluded if they focused solely on the development of technical specifications of the technology without reporting on clinical outcomes, did not report hospitalization rates or total number of hospitalizations as an outcome, did not include populations considered to be medically underserved, or were not available in full text. Additionally, studies that were solely literature review in nature had their bibliographies manually reviewed for relevant articles but were not included. 

A multi-level screening process was utilized to identify studies to be included in the review. A single reviewer independently screened all titles and abstracts for potential inclusion initially. Full-text articles were retrieved and assessed for eligibility based on the inclusion and exclusion criteria after assessment of the initial abstract and title review. Uncertainty regarding eligibility was resolved by consulting with a second subject matter expert when necessary. Data was then standardized to extract patient population size, hospitalizations, type of wearable technology, specialty of the article, country of publication, and patient population location. 

After inclusion and exclusion were confirmed, the studies were then stored within Endnote for review and data extraction. Hospitalizations, patient demographics, and comorbidity information were extracted as either a raw number of hospitalizations or as the rate of hospitalizations within the group. Hospitalization rates were recorded as a percent of the population total. If a total number of hospitalizations was provided and a rate of hospitalizations was not provided, then the hospitalization rates were calculated using the formula of a total number of hospitalizations divided by the total population of the study. All statistics were then calculated using the minimum, maximum, and average percent of criteria across all studies. After data extraction, a narrative synthesis of results was tabulated using Microsoft Excel (Microsoft Corp., Redmond, WA) to generate the primary statistics as well as any additional subanalyses.

Primary evaluation factors included the type of technology used, the medical specialty, and geographical characteristics within each study. Hospitalization rates were then compared within and between studies to evaluate the patterns in outcomes based on technology type, region, specialty of disease, year, and year in relation to the COVID-19 pandemic. The risk of bias was assessed using the Cochrane risk of bias tool for randomized controlled trials (RCTs). All other studies were assessed for risk using the Critical Appraisal Skills Program (CASP) cohort study checklist.

Results

Study Selection

**Figure 1:**
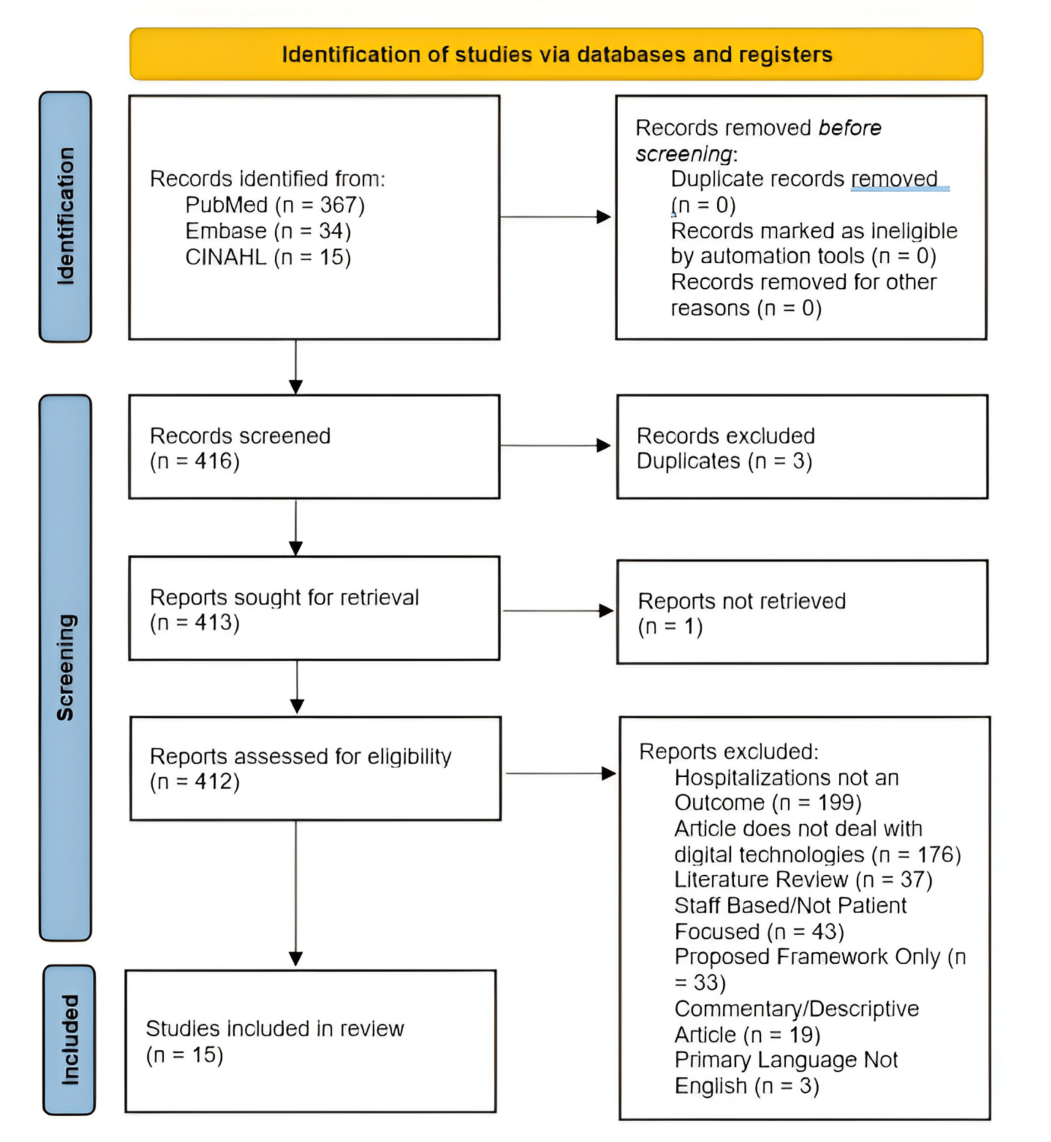
PRISMA flow diagram illustrating the study selection process

After using the search terms in each database, a total of 416 articles were found to meet the search query. All articles were screened and reviewed based on the criteria discussed previously in this review. Of these 416 articles, 14 were selected for review because they met the criteria (n=14) (Figure 1) (Table 1).

**Table 1:** Selected study description MORES: mobile obstetric emergency system; DRC: Democratic Republic of the Congo; ED: emergency department; PCPs: primary care providers; TVC: telehealth videoconferencing; RCT: randomized controlled trial, SINEMA: primary care-based integrated mobile health; ENABLE CHF-PC: Educate, Nurture, Advise, Before Life Ends Comprehensive Heartcare for Patients and Caregivers; GDMT: guideline-directed medical therapy

Article number	Year written	Primary author's last name	Title	Study type	Device type	Device subtype/name	Study objective	Primary outcomes	Secondary outcomes	Hospitalization outcome primary or secondary	Hospitalization measure
1	2024	Lee [8]	The use of a mobile obstetric emergency system to improve obstetric referrals in Bong County, Liberia: a pre-post study	Pre-post study	Telehealth		to examine the association between MORES implementation and referral time for obstetric emergencies as well as maternal/newborn outcomes	maternal outcome, newborn outcome, transfer time from rural health facility to district hospital		Primary	Percentage of the population with hospital transfers of at least 2 hours and at least 12 hours
2	2020	Bakitas [9]	Effect of an early palliative care telehealth intervention vs usual care on patients with heart failure: the ENABLE CHF-PC randomized clinical trial	RCT	Telehealth	ENABLE CHF-PC and telephone	To determine the effect of a 16-week early palliative care telehealth intervention on the quality of life, mood, global health, pain, and resource use in patients with advanced heart failure	Quality of life measures (Kansas City Cardiomyopathy Questionnaire)	Global patient health, pain, hospital days, and ED visits	Secondary	Days in hospital last 0/8/16 weeks and ED visits last 0/8/16 weeks
3	2024	Eberly [10]	Telephone-based guideline-directed medical therapy optimization in Navajo Nation: the hózhó randomized clinical trial	RCT	Telehealth		To understand if a phone-based telehealth model for heart failure with reduced ejection fraction increases uptake of guideline-directed medical therapy	Number of guideline-directed classes of drugs filled from the pharmacy	Increase each individual class of GDMT, cardiac referrals made, cardiac referrals completed, cardiac procedures or interventions, heart failure hospitalizations	Secondary	Number/percent of population with hospital admission
4	2021	Diaka [11]	Leveraging smart glasses for telemedicine to improve primary healthcare services and referrals in a remote rural district, Kingandu, DRC, 2019–2020	Observational	Smart glasses	Iristick smart glasses	To improve primary healthcare services, especially referrals to the district hospital, for the population in three health centers in the rural district of Kingandu in the DRC by introducing smart glasses and leveraging them for telemedicine	Average Monthly Consultations and Hospital Referrals		Primary	Number of Referrals to Hospital
5	2016	Frail [12]	Experience with technology-supported transitions of care to improve medication use	Observational	Telehealth		To describe an innovative community pharmacy-based program using technology to support transitions of care for patients, particularly those residing in rural areas	Hospital readmission rates		Primary	Frequency of hospital readmission
6	2019	Bian [13]	Association of a school-based, asthma-focused telehealth program with emergency department visits among children enrolled in South Carolina Medicaid	Retrospective analysis	Telehealth		To examine the associations of a school-based telehealth program in Williamsburg County (South Carolina) with all-cause ED visits made by children enrolled in Medicaid	Status of at least 1 all-cause ED visit by a child in a given month		Primary	Mean proportion of ED visits
7	2018	Jang [14]	Impact of a wearable device-based walking program in rural older adults on physical activity and health outcomes: cohort study	Population-based prospective cohort study	Mobile app	Smart walk	To identify whether wearable devices improve physical activity and health outcomes in older adults in rural areas, whether there are differences in physical activity and health outcome improvements depending on frailty status, and whether there are differences in wearable device adherence between coaching and self-management	Usual gait speed, IPAQ score, BMI, and total body fat mass	Number of falls, number of outpatient days, and number of ER days	Secondary	Number of inpatient admission days and number of ER days
8	2024	Jones [15]	Cellular-enabled remote patient monitoring for pregnancies complicated by hypertension	Mixed methods: pre-post survey design and semi-structured qualitative interview	Remote patient monitoring	BodyTrace, including blood pressure cuff and weight scale. Cuff equipped with cellular transmission capabilities	To assess maternal and neonatal clinical outcomes as well as patient acceptability of an integrated model of cellular-enabled RPM devices for BP supported by a 24/7 nurse call center	Perceived stress, anxiety, perceived benefits, system usability and satisfaction, and behavioral intervention	Hospitalizations and ED visits	Secondary	Total number of hospitalizations, and mean proportion of hospitalizations
9	2022	Somers [16]	Enhanced telemedicine in a mobile integrated health program to improve healthcare access during the COVID-19 pandemic	Retrospective chart review	Telehealth		To have patients connect with their PCPs shortly after discharge so that the PCPs can continue to manage their chronic health conditions and medications and prevent readmission to the hospital	Readmission rate		Primary	Number/percent of population readmitted to hospital (risk-adjusted and not risk-adjusted)
10	2021	Lambooy [17]	Telemedicine for outpatient care of kidney transplant and CKD patients	Longitudinal observational cohort study	Telehealth		To assess the feasibility, sustainability, and clinical outcomes of TVC for patients with chronic kidney disease or kidney transplant recipients	Feasibility	Change in creatinine, eGFR, systolic/diastolic blood pressure, overnight hospital admission days, and travel distance to tertiary hospital outpatient clinic	Secondary	Number of hospitalizations
11	2018	Martinez [18]	mHealth intervention to improve the continuum of maternal and perinatal care in rural Guatemala: a pragmatic, randomized controlled feasibility trial	Pragmatic feasibility trial	Mobile app	mHealth	To characterize baseline rates of complication detection and facility-level referral in rural Guatemala, and to evaluate the impact of the mHealth system on these rates	Number of monthly referrals to facility-level care from TBA's for maternal and perinatal complications, adjusted by the monthly birth volume	Proportion of referrals completed	Primary	Adjusted monthly emergency referral rate per 100 births
12	2021	Yan [19]	Effectiveness of a primary care-based integrated mobile health intervention for stroke management in rural China (SINEMA): a cluster-randomized controlled trial	RCT	Mobile app		To determine whether a SINEMA intervention could improve stroke management in rural China	12-month change in systolic blood pressure	Diastolic blood pressure, mobility functioning, physical activity, health-related quality of life, self-reported medication adherence to antiplatelet therapy, stroke recurrence, hospitalization, disability, and mortality	Secondary	Number/percent of population with stroke hospitalization
13	2022	Cooper [20]	Evaluation of myCOPD digital self-management technology in a remote and rural population: real-world feasibility study	1 year longitudinal test of change evaluation	Mobile app	myCOPD	To evaluate myCOPD and its effectiveness at reducing hospitalizations, inpatient bed days, and other health service use	Inpatient bed days, hospital admissions, home visits, clinic appointments, and out of hours care provision		Primary	
14	2024	Cohen [21]	Newborn readmissions and virtual primary care delivery: a population-based case-control study	Case-control study	Telehealth		To understand whether primary care visit modality (in-person vs. virtual) is associated with early newborn hospital readmissions and ED visits	Newborn readmission within the first 14 days of life by primary care visit modality		Primary	Number/percent of patients with readmission who were assessed through telehealth vs. in-person visits
15	2019	Locke [22]	Using video telehealth to facilitate inhaler training in rural patients with obstructive lung disease	Retrospective chart review	Telehealth	Clinical video telehealth to home	Pilot program for rural patients who received inhaler training education sessions at home from a pharmacist over video telehealth	Teach-to-goal scores between initial visit and third visit and patient satisfaction	Exacerbations	Secondary	Number of exacerbations

The primary reasons for articles not being selected for review included hospitalizations not being an outcome within the article itself (n=199), the article not handling telehealth or remote biometric technology (n=176), the articles being concerned with frontline staff burnout rates rather than patient hospitalization rates (n=43), and the retrieved article being only a proposed framework or study design without data (n=33). Additionally, several articles pertained to the use of telemedicine in a hospital stroke setting, but since these focused on the effect of utilizing telehealth with already hospitalized patients rather than hospitalization rates, they were excluded (n=8). Three duplicate articles were identified between the databases and excluded during manual review and retrieval (n=3). 

Additionally, several literature reviews were identified utilizing the search criteria (n=37). These literature reviews were excluded, but the bibliographies of each literature review were screened for potential additional articles. This process resulted in one additional article being included within the study selection for meeting criteria (n=15).

Study Characteristics

**Figure 2:**
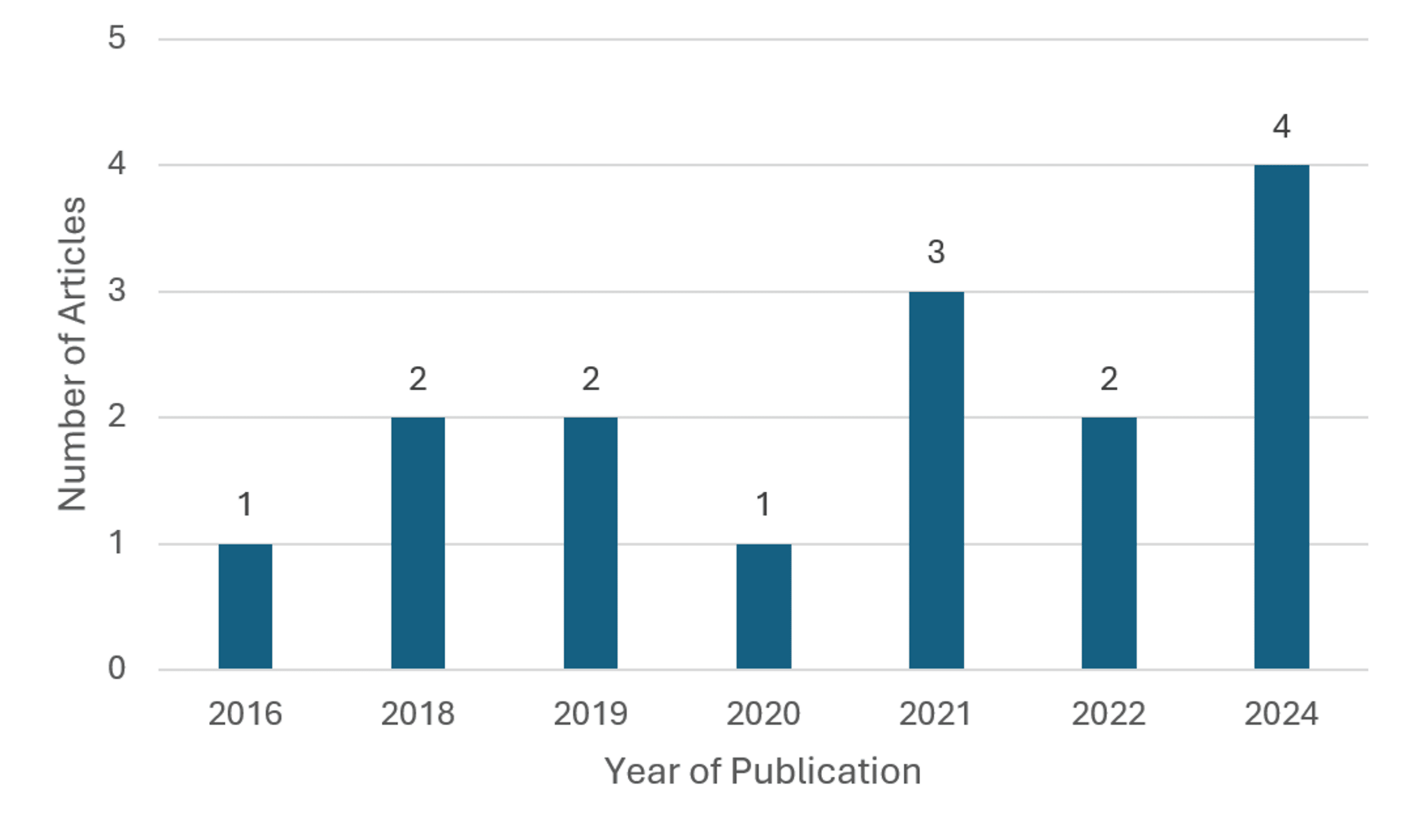
Number of articles retrieved by year

**Figure 3:**
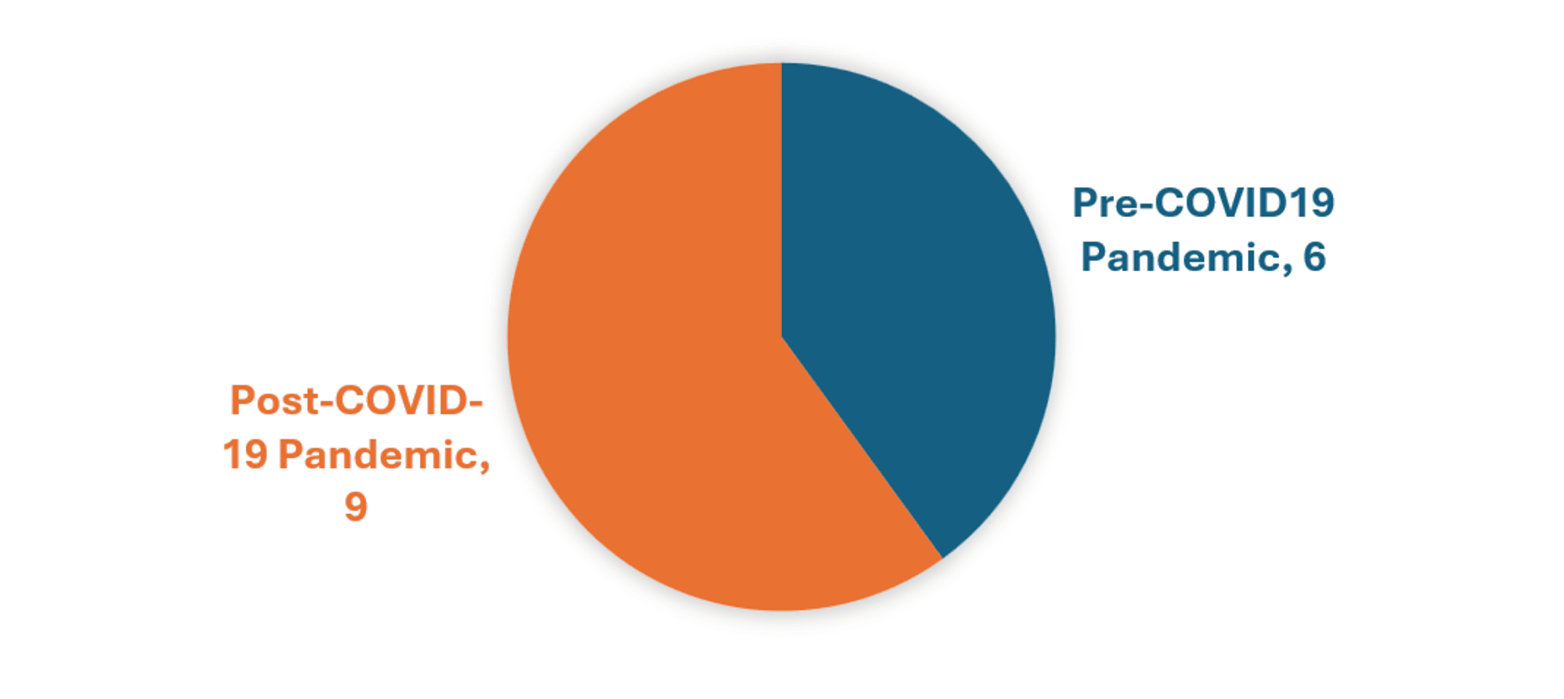
Studies performed in relation to COVID-19 pandemic

Most of the articles were written after or during the COVID-19 pandemic. Of the 15 articles, 60% were written between 2021 and 2024, while 33% were written between 2016 and 2019 (Figure 2). One article was written in 2020 [9]. Research for this article was conducted between 2015 and 2019 in patients with heart failure, a high proportion of whom were from rural and African American populations. As the research was performed prior to COVID-19, the article was included with the pre-COVID-19 studies. However, its publication date in September may suggest that it should be grouped with the post-COVID-19 article (Figure 3).

There were three RCTs identified (n=3), with the rest being observational studies (n=12). Of the observational studies, retrospective chart reviews (n=2) and pre-post studies (n=2) were the most common types. Two retrospective chart reviews, two RCTs, a retrospective analysis, one observational study, and a mixed methods study were performed within the United States (n=7).

**Figure 4:**
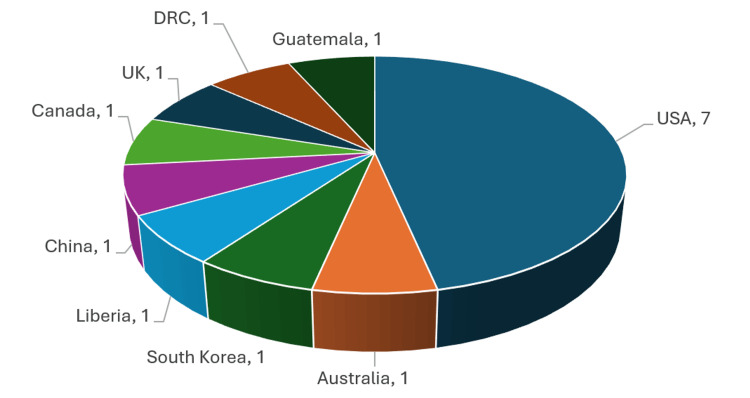
Selected studies by country of origin DRC: Democratic Republic of the Congo

The most studied technologies were telehealth visits (n=9), while mobile apps (n=4), smart glasses (n=1), and remote patient monitoring devices (n=1) made up the remaining technologies studied. The remote patient monitoring study was performed in the United States, mobile app studies were performed in China (n=1), Guatemala (n=1), South Korea (n=1), and the United Kingdom (n=1), and the smart glasses study was performed in the DRC (n=1) (Figure 4).

**Figure 5:**
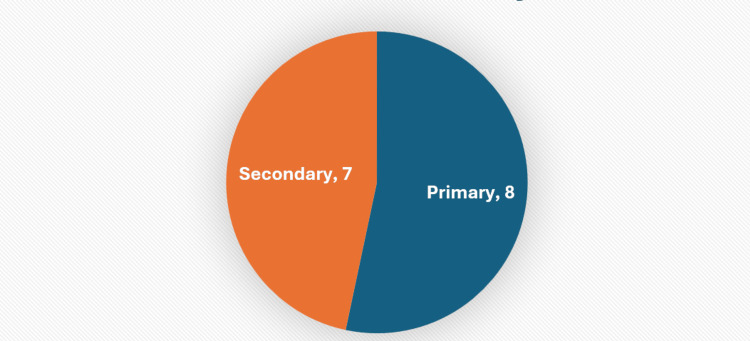
Articles by primary hospitalization outcome

Hospitalizations were more commonly taken as a primary outcome as opposed to a secondary outcome, although this was not significant. Eight of the articles evaluated had hospitalizations as a primary outcome (Figure 5). One of these articles evaluated rural populations in Bong County in Liberia. This study used a pre-post study design to evaluate the association between their mobile obstetric emergency system implementation and referral time for obstetric emergencies. This was in place of inpatient days or number of ED visits [8]. Their hospitalization measure was, instead, the percent of the population with transfer to hospital times of at least two hours and at least 12 hours. The researchers here found no statistically significant difference in transfer time between rural health facilities to hospitals between baseline and end line [8].

Risk of Bias

Risk of bias analysis was completed using the Cochrane Risk of Bias tool for the three studies described as RCTs and the CASP checklist for the observational studies. Two studies showed a high risk of bias, while one showed a low risk of bias (Table 2). Allocation concealment and performance bias were the areas most at risk; in “Effect of an early palliative care telehealth intervention vs usual care on patients with heart failure: the ENABLE CHF-PC randomized clinical trial” [9] and “Telephone-based guideline-directed medical therapy optimization in Navajo Nation: the hózhó randomized clinical trial” [10], the patients and clinicians were not blinded to their randomization, which introduced a fairly high risk of bias. Additionally, in the Educate, Nurture, Advise, Before Life Ends Comprehensive Heartcare for Patients and Caregivers (ENABLE CHF-PC) trial, there was an unusual risk of bias in that "all participants received a $10 incentive at each data collection time point" [9].

**Table 2:** Risk of bias assessment (randomized controlled studies using Cochrane tool)

Article number	Year written	Primary author's last name	Title	Selection bias (random sequence generation)	Selection bias (allocation concealment)	Reporting bias	Performance bias	Detection bias	Attrition bias	Other sources of bias	Risk of bias
2	2020	Bakitas [9]	Effect of an early palliative care telehealth intervention vs usual care on patients with heart failure: the enable CHF-PC randomized clinical trial	Low	High	Low	High	High	Low	High	High
12	2021	Yan [19]	Effectiveness of a primary care-based integrated mobile health intervention for stroke management in rural China (Sinema): a cluster-randomized controlled trial	Low	Low	Low	Medium	Low	Low	Low	Low
3	2024	Eberly [10]	Telephone-based guideline-directed medical therapy optimization in Navajo Nation: the hózhó randomized clinical trial	Unclear	High	High	High	Low	Low	Low	High

Additionally, bias was assessed using the CASP checklist modified for cohort studies to assess the remaining observational studies. (n=12) Of the remaining studies, the majority did not show a substantial risk of bias (n=10). However, two studies did demonstrate a risk of bias due to issues within the study design (Table 3) [11,12].

**Table 3:** Risk of bias assessment (randomized controlled studies using CASP cohort study checklist) CASP: Critical Appraisal Skills Program

Index	Year	Author	Title	Did the study address a clearly focused issue?	Was the cohort recruited in an acceptable way?	Was the exposure accurately measured to minimize bias?	Was the outcome accurately measured to minimize bias?	Have the authors identified all-important confounding factors?	Have they taken account of the confounding factors in the design and/or analysis?	Was the follow-up of subjects complete enough	Was the follow-up of subjects long enough?	What are the results of this study	How precise are the results?	Do you believe the results?	Can the results be applied to the local population?	Do the results of this study fit with other available evidence?	What are the practical implications of this study for practice?	Risk of bias determination
1	2024	Lee [8]	The use of a mobile obstetric emergency system to improve obstetric referrals in Bong County, Liberia: A pre-post study	Yes	Yes	Yes	No	No	No	No	No	Yes	Yes	Yes	No	Yes	Yes	No substantial risk of bias
4	2021	Diaka [11]	Leveraging smart glasses for telemedicine to improve primary healthcare services and referrals in a remote rural district, Kingandu, DRC, 2019–2020	Yes	Yes	No	Yes	No	No	No	No	Yes	No	Yes	No	Yes	No	Risk of bias
6	2019	Bian [13]	Association of a school-based, asthma-focused telehealth program with emergency department visits among children enrolled in South Carolina Medicaid	Yes	Yes	Yes	Yes	Yes	Yes	No	Yes	Yes	Yes	Yes	Yes	Yes	Yes	No substantial risk of bias
7	2018	Jang [14]	Impact of a wearable device-based walking programs in rural older adults on physical activity and health outcomes: cohort study	Yes	Yes	Yes	Yes	No	Yes	Yes	Yes	Yes	Yes	Yes	Yes	Yes	Yes	No substantial risk of bias
8	2024	Jones [15]	Cellular-enabled remote patient monitoring for pregnancies complicated by hypertension	Yes	Yes	Yes	Yes	No	Yes	No	Yes	Yes	No	Yes	Yes	No	No	No substantial risk of bias
9	2022	Somers [16]	Enhanced telemedicine in a mobile integrated health program to improve healthcare access during COVID-19 pandemic	No	Yes	Yes	No	No	No	Yes	No	Yes	No	Yes	Yes	Yes	Yes	No substantial risk of bias
10	2021	Lambooy [17]	Telemedicine for outpatient care of kidney transplant and CKD patients	Yes	Yes	Yes	Yes	Yes	Yes	Yes	Yes	No	Yes	No	Yes	No	Yes	No substantial risk of bias
11	2016	Frail [18]	Experience with technology-supported transitions of care to improve medication use	No	Yes	No	No	No	No	Yes	No	Yes	Yes	Yes	Yes	No	No	Risk of bias
11	2018	Martinez [19]	mHealth intervention to improve the continuum of maternal and perinatal care in rural Guatemala: a pragmatic, randomized controlled feasibility trial	Yes	Yes	Yes	Yes	Yes	Yes	Yes	Yes	Yes	Yes	Yes	Yes	Yes	Yes	No substantial risk of bias
13	2022	Cooper [20]	Evaluation of myCOPD Digital Self-management Technology in a Remote and Rural Population: Real-world Feasibility Study	Yes	Yes	Yes	Yes	No	Yes	Yes	Yes	No	Yes	Yes	Yes	Yes	Yes	No substantial risk of bias
14	2024	Cohen [21]	Newborn readmissions and virtual primary care delivery: a population-based case-control study	Yes	Yes	Yes	Yes	Yes	Yes	No	No	Yes	Yes	Yes	Yes	No	Yes	No substantial risk of bias
15	2019	Locke [22]	Using video telehealth to facilitate inhaler training in rural patients with obstructive lung disease	Yes	Yes	No	Yes	Yes	Yes	No	No	Yes	No	Yes	No	Yes	No	No substantial risk of bias

Population Characteristics

Populations described in the articles varied considerably. In evaluating the size of the studies, the experimental arm tended to have fewer patients (Avg.=1064.6) than the control arms of each study (Avg.=15996.6) (Table 4). Demographic information was more readily available in articles written in the United States, while articles from outside the United States often lacked demographic information. Of the articles that included demographic information, patients in the control arm tended to be Caucasian more frequently (Avg.=41.9%) than in the experimental arm (Avg.=28.7%). Additionally, within the United States, experimental arm subjects were more likely to be African American (Avg.=73%) compared to the control arm (Avg.=54.6%). Several studies dealt with pediatric patients as opposed to adult populations. In the adult populations, ages were normally given as age bands rather than as mean age. Of the adult population studies, patient ages tended to be older in the control arm (Avg.=65.7) as opposed to the experimental arm (Avg.=65.3). Additionally, patients in the experimental arm reported more frequently had a lower level of education than those in the control arm and were more likely to have an education level above primary school (Avg.=68.7%) compared to the control arm as well (Avg.=67.6%). Selected study populations were predominantly from rural communities (n=11), while Indigenous (n=3) and low-income (n=1) populations were also included. 

**Table 4:** Patient demographics by selected criteria COPD: chronic obstructive pulmonary disease; OSA: obstructive sleep apnea; GERD: gastroesophageal reflux disease

Demographic criteria	Experimental	Control
Articles reporting total patients	15/15 (100%)	9/15 (60%)
Min, max (avg.)	11-4043 (1064.6)	4-38438 (15996.6)
Articles reporting % Caucasian	5/15 (33.3%)	4/15 (26.7%)
Min, max (avg.)	8-100 (73)	4-38438 (15996.6)
Articles reporting % African American	3/15 (20%)	2/15 (13.3%)
Min, max (Avg.)	54.3-80.9 (73)	4-38438 (15996.6)
Articles reporting % other race	6/15 (40%)	3/15 (20%)
Min, max (avg.)	1.4-100 (47)	1-15.7 (10.3)
Articles reporting gender rate	10/15 (66.7%)	6/15 (40%)
Min, max (avg.) (% male)	18.6-18.6 (73)	50.5-100 (57.7)
Comorbidities reported	7/15 (46.7%)	0/15 (0%)
CAD	21-57 (43.2)	N/A
COPD	11-100 (68.1)	N/A
Heart failure	77-96 (83.8)	N/A
CKD	24-43 (34.5)	N/A
Asthma	3-10.2 (8)	10.5-11.4 (10.9)
GERD	1-5 (2)	1.8-2 (1.9)
Obesity	1.7-3.3 (2.3)	2.9-3.7 (3.2)
OSA	0.4-2.1 (0.9)	0.6-2.5 (1.3)
Sinusitis	4.5-8.3 (6.4)	6.5-9.7 (7.9)
Anxiety/depression/emotional disturbance	2.2-7.4 (4.2)	4.1-5.7 (4.9)
Hypertension	0.4-72.4 (26.7)	0.5-65.9 (10.2)
Diabetes	0.3-71 (27.3)	0.4-15.6 (3.1)
Articles reporting education level	4/15 (26.7%)	4/15 (26.7%)
Less than high school	10-100 (77.1)	10-100 (74)
Above primary school	30-90 (68.7)	27.6-90 (67.6)
High school/GED	30-39.4 (34.7)	23.7-30 (26.9)
Some college	29.3-45 (37.2)	40.6-45 (42.8)
College graduate	13.9-15 (14.5)	12.6-15 (13.8)
Graduate degree	3.4-3.4 (3.4)	8.2-8.2 (8.2)

Study Outcomes

Hospitalizations were grouped as either inpatient hospitalizations (n=8), ED visits (n=4), or other hospital referrals in cases where a patient may be referred to a hospital but was not classified as either inpatient hospitalization or emergency (n=7). Across the emergency group studies, the experimental arm of the studies had a lower rate of ED visits (Avg.=21.2%, n=4) compared to the control arm of these studies (Avg.=25.7%, n=3) (Table 5) [13-15]. Similarly, inpatient hospitalizations showed a decrease in hospitalization rates when comparing the control (Avg.=42.3%, n=4) and intervention arms (Avg.=15.6%, n=8) (Table 5) [10,15-20]. However, not all of the studies had both an intervention and control arm. When controlling for studies that included both control and experimental arms, inpatient hospitalization rates in the experimental arm remained (Avg.=31.9%, n=4) compared to the control arm (Table 6) [10,17-19]. One study did not report any hospitalizations or ED visits, despite tracking this as a secondary outcome [17].

**Table 5:** Outcomes by study experimental group arm versus study control group arm ED: emergency department

Outcome	Experimental	Control
Articles reporting ED visit rate	4/15 (26.7%)	3/15 (20%)
Min, max (avg.) (visits/patient (%))	0-111.1 (21.2)	3.27-160 (25.7)
Articles reporting inpatient admissions	8/15 (53.3%)	4/15 (26.7%)
Min, max (avg.) (visits/patient (%))	0-100 (15.6)	0-160 (42.3)
Articles reporting other hospital referral metrics	7/15 (46.7%)	3/15 (20%)
Min, max (avg.) (visits/patient (%))	4.4-100 (30.5)	3.3-21.6 (11.4)

**Table 6:** Outcomes by study experimental group arm versus study control group arm in studies have both control and experimental groups ED: emergency department

Outcome	Experimental	Control
Articles reporting ED visit rate	3/15 (20%)	3/15 (20%)
Min, max (avg.) (visits/patient (%))	0-111.1 (16.7)	3.27-160 (25.7)
Articles reporting inpatient admissions	4/15 (26.7%)	4/15 (26.7%)
Min, max (avg.) (visits/patient (%))	0-100 (31.9)	0-160 (42.3)
Articles reporting other hospital referral metrics	3/15 (33.3%)	3/15 (20%)
Min, max (avg.) (visits/patient (%))	4.4-100 (36.6)	3.3-21.6 (11.4)

Outcomes Subanalysis

Multiple subanalyses were performed, including the rates of hospitalizations for telehealth visits only, the rates of hospitalizations for non-telehealth visits, comparative hospitalization rates between telehealth versus other interventions, analysis of the other hospitalization rates not classified as inpatient admission or ED visits, and multi-cohort studies.

Within the articles discussing telehealth as an intervention, the studies discussing telehealth were more likely to discuss rates of inpatient hospitalization rates (n=4) than ED visit rates (n=1). The experimental arm of these studies demonstrated higher rates of inpatient admissions (Avg.=2.7%, n=4) than the control groups (Avg.=0%, n=1) (Table 7) [8,11,12,15,17].

**Table 7:** Outcomes by study experimental group arm versus study control group arm in telehealth studies only ED: emergency department

Outcome	Experimental	Control
Articles reporting ED visit rate	2/15 (13.3%)	1/15 (6.7%)
Min, max (avg.) (visits/patient)	3.6-57.1 (11.4)	3.2-3.9 (3.5)
Articles reporting inpatient admissions	4/15 (26.7%)	1/15 (6.7%)
Min, max (avg.) (visits/patient)	0-12.5 (2.7)	0/0 (0)
Articles reporting other hospital referral metrics	4/15 (26.7%)	1/15 (6.7%)
Min, max (avg.) (visits/patient)	7.7-100 (36.8)	21.6-21.6 (21.6)

The non-telehealth study subanalysis demonstrated similar decreases in both ED and inpatient admission rates. The experimental arm’s ED visit rate (Avg.=55.6%, n=2) favorably compared to the control arm (Avg.=92.5%, n=2), similar to the inpatient admission rate in both experimental (Avg.=33.7%, n=4) and control arms (Avg.=56.4%, n=3) (Table 8) [10,12,14,15,19,20].

**Table 8:** Outcomes by study experimental group arm versus study control group arm in non-telehealth studies ED: emergency department

Outcome	Experimental	Control
Articles reporting ED visit rate	2/15 (13.3%)	2/15 (13.3%)
Min, max (avg.) (visits/patient)	0-111.1 (55.6)	25/160 (92.5)
Articles reporting inpatient admissions	4/15 (26.7%)	3/15 (20%)
Min, max (avg.) (visits/patient)	4.4-100 (33.7)	0-160 (56.4)
Articles reporting other hospital referral metrics	3/15 (20%)	2/15 (13.3%)
Min, max (avg.) (visits/patient)	4.4-39.1 (24.1)	3.3-9.3 (6.3)

Of these non-telehealth studies (n=5), subanalysis showed that there were four mobile app studies [14,18,19,20]. Of the non-telehealth studies, one of the studies discussed hospitalizations using other means. This study was about the use of smart glasses technologies and discussed the rate at which referrals from the rural health center in Kingandu, DRC, were made to the larger hospital center. This study found that the utilization of smart glasses technology improved referral to hospital rates from 3.3% of visits to the health center to 39.1% [11].

Of the selected studies, 10 (n=10) studies had disease-specific inclusion criteria. The most common disease-specific inclusion criteria were pregnancy (n=3) and pulmonary diseases such as asthma or COPD (n=3), with heart failure (n=2) being the third most common. 

Seven (n=7) studies had disease-specific hospitalization measures. The most common conditions evaluating condition-specific hospitalizations were for pregnancy (n=3), followed by obstructive lung disease (n=2). Another study reviewing the efficacy of utilizing a mobile app in rural China provided data regarding stroke rehospitalizations over the past year; this study found that stroke rehospitalizations improved from 9.3% of the population to 4.4%, while another captured the frequency of heart failure hospitalizations within the Navajo nation in the United States; this study showed fewer heart failure hospitalizations in their intervention arm (1.3%) compared to usual care (4.3%) [10,19]. Of the pregnancy studies, one of the studies specifically utilized a remote patient monitoring device to monitor pregnant patients who had pregnancies complicated by hypertension in rural Arkansas, United States [15]. The remainder of the studies (n=8) were captured on all-cause ED visits or inpatient admission rates. The disease-specific rehospitalization rates were identified as hospitalization rates, within our dataset, but with the caveat that this does not represent an all-cause hospitalization rate.

A final study, not discussing telehealth, examined the use of remote patient monitoring technologies in pregnancies complicated by hypertension, utilizing a blood pressure cuff with transmission capabilities and a weight scale [15]. In this study, the authors provided a usual care hospitalization rate of 4.3%, while the experimental arm patients utilizing the technologies had a total of 20 ED visits and 20 inpatient hospitalizations with a mean ED visit rate of 0.75 visits per patient, and a mean inpatient hospitalizations of 0.35 hospitalizations per patient. 

Comparison between telehealth and non-telehealth wearable technologies such as mobile apps or remote blood pressure cuffs remains difficult. When accounting for the above, telehealth experimental arm patients had a reduced inpatient hospitalization rate (Avg.=2.7%, n=4) compared to other interventions inpatient hospitalization rates (Avg.=13.9%, n=4) [8,11,12,15]. However, since only one study provided the inpatient hospitalization rate, compared to the three studies that provided this data for telehealth, the results here may have limited comparative utility.

There were two United States-based telehealth studies where data was provided over multiple cohorts beyond the standard control/intervention arms. One study evaluated the effect of a school-based, asthma-focused program among South Carolina Medicaid patients within the state of Williamsburg, SC [13]. In this study, the authors evaluated the effects of pre- and post- implementation of a school-based asthma program within South Carolina based on Medicaid claims data. The authors also compared the effect of these programs to neighboring counties that were less underprivileged. In this study, data was provided between the years of 2012 and 2017, with the years of 2012-2014 being considered “pre-intervention” while 2015-2017 were “post-intervention.” Within this study, it was found that the experimental arm (i.e., the Williamsburg arm) showed an increase in ED visit rates, from 3.65% of students to 3.87% of students. A similar increase in ED visits was found within the control arm of the study as well (3.37%/3.56%). Based on this, the analysis demonstrated a 21% relative decrease in ED visits per month in children with asthma with the implementation of the program. 

A second United States-based telehealth study providing data over multiple cohorts discussed the effect of a telephone-based medical therapy optimization in the Navajo nation among heart failure patients [10]. In their experimental model, heart failure patients at least 18 years of age or older with left ventricular EF of 40% or less, a primary care encounter, and a clinical encounter at an Indian Health Service (IHS) site with a prescription in the last 12 months were included in a stepped trial study where patients were initially provided with increasing interventions until all cohort patients were utilizing the full telehealth model. Hospitalizations in this model were a secondary outcome, and fewer heart failure hospitalizations were identified in the intervention arm as opposed to the usual care arm [10].

There was one article that evaluated the frequency with which newborns were readmitted based on the modality through which they were seen by primary care after discharge. In the sole Canadian study evaluating patients in Ontario, Canada, cases of neonatal readmission were compared to both a matched cohort and an unmatched cohort of neonates without readmission within 14 days of life [21]. Here, it was found that neonates who had a virtual visit within the first seven days of life were more likely to have hospital readmission than those in the matched controls; 3.9% of neonatal readmissions had a virtual visit within seven days of life as opposed to 3.1% of matched controls having a virtual visit within seven days of life. In this study, the rates were reversed and not as comparable as given. However, when reversing the data to evaluate how many patients had a virtual visit within the first seven days of life, we find that 23.6% of neonates who had a virtual visit within the first seven days of life had readmission compared to 19.8% of neonates who had an in-person visit. Similarly, 27.3% of neonates with a virtual visit within 10 days of life experienced readmission compared to 19.8% of neonates who had an in-person visit. This represents an increased risk of readmission among neonates who have a virtual visit as opposed to an in-person visit, which could be attributed to a more limited physical examination; jaundice was the most common reason for readmission overall 69.8% of readmissions in the virtual group, 71.6% of admissions in in-person, and infectious admissions being more frequent in the virtual group (11.6% virtual group, 7.4% in-person group) [21].

A final article to consider was an article written to evaluate the use of video telehealth to improve outcomes among rural patients with obstructive lung diseases, such as COPD or asthma [22]. This study did not provide a true number of hospitalizations per se but did characterize the total number of acute exacerbations of the patient. While the terminology utilized within the text indicates that these could potentially be considered hospitalizations, these instances were considered other hospital referrals. The authors of this study did not note any significant difference in pre- and post- training hospitalization rates [22].

Discussion

Since the COVID-19 pandemic, health professionals have rapidly had to adapt to a changing world, with new and developing technologies at their disposal. For example, how can health professionals accurately diagnose or monitor patients remotely without traditional physical examinations? When considering that 19.3% of the United States population lives in rural areas, according to the United States Census, and the migration from urban to rural areas that occurred, in part, due to the pandemic, it is more important than ever for healthcare professionals to find ways to deliver care to these potentially underserved areas [23,24]. Digital technologies could significantly help optimize patient care, thereby reducing hospitalizations in these underserved areas.

However, there still appears to be some resistance within healthcare circles to evaluating the effectiveness of certain new technologies that may provide overall healthcare benefits for patients while also affecting healthcare utilization rates. Despite the availability of a variety of technologies to evaluate patients remotely, the technology that appeared to be most studied during the course of this literature review was telehealth technologies. Sixty percent of the articles found in the course of the review primarily focused on telehealth and remote visit technologies, as opposed to patient monitoring devices or other mobile applications that could be used to evaluate patient health conditions before they go to an acute care center for evaluation [8-10,12-13,16-17,21-22]. By comparison, only 40% of the articles focused on technologies other than telehealth visit technologies [11,14-16,18-20].

Context and limitations

As with any emerging technology, there are complications involved in evaluating how these new digital technologies might affect a patient’s healthcare utilization rate. One such variable may be the level of education, as evidence exists that adults with higher levels of education live longer and healthier lives compared to less educated peers [25]. In rural and underserved populations, the level of education could be a confounding factor for the utilization of these new technologies. In the United States, for example, 45% of persons in rural areas are likely to have the highest level of education of high school or less compared to 35% of persons in suburban areas or 36% of persons living in city areas [26]. Even outside the United States, people living in these underserved areas may not have access to the same level of education as those living in more populated areas; for example, a study in rural India indicated a 65.4% illiteracy rate [27]. An example here is seen in “Effectiveness of a primary care-based integrated mHealth intervention for stroke management in rural China (SINEMA): A cluster-randomized controlled trial,” wherein the intervention group was more likely to have above primary school education (30%) compared to the control group (27.6%) [19]. Similarly, in “Effect of an early palliative care telehealth intervention vs usual care on patients with heart failure: the ENABLE CHF-PC randomized clinical trial”, intervention subjects were more likely to have at least a high school education compared to those in the usual care group. However, this was not statistically significant [8]. As such, to utilize these new technologies, these barriers may act as a floor, which would prevent patients from accessing care utilizing these new technologies. 

However, it is important for us to consider long-term patient treatment in addition to potential short-term gains. Certain studies, such as the SINEMA study, had a limited duration of 12 months may not provide us with an idea of long-term hospitalization rates for this population, leading us to need to consider how telehealth visits may improve patient long-term care as opposed to short-term gains [19]. Long-term studies on the effect of telehealth and other mHealth interventions, such as the South Carolina asthma study, will be required for us to better assess patient care outcomes using these technologies [13].

Costs may be an additional limiting factor for providing these technologies in rural areas. Per capita income in the United States was $64,143 across all Americans or ~$1,233.52 weekly. This income rate dropped to $49,895 per capita in rural areas or ~$959.52 weekly [28]. A Fitbit Versa 2 Fitness Smartwatch can cost upwards of $149 at Walmart [29]. While, for the average American, this Smartwatch may cost only 12% of their weekly income, this would increase to ~15.5% of the average rural American’s income. We see this effect in studies such as “Telemedicine for outpatient care of kidney transplant and CKD patients”, wherein 75% of the control CKD group was found to have an income of less than $30k AUD, compared to 37.5% of the telemedicine group. While less pronounced, 68.75% of the control KTR group in this study had an annual income of less than $60k AUD, compared to only 50% of the intervention group [17]. When combined with the fact that residents of rural areas are more likely to lack health insurance compared to more urbanized regions, this does demonstrate how rural areas may lack the resources to access these devices, which could potentially improve patient health outcomes [30].

Additionally, there exists a possibility that this area may not be as well researched in underserved populations as in other populations. The search terms without any filtering discussed in the Methods section provided us with 303 results from PubMed since 2015, 20 of which had an article type of clinical trial or RCT. By comparison, if we leave off the medically underserved part of the search equation, we instead find 3170 results for clinical trial and RCT only, representing a substantial difference in the level of research into how to best provide care to these underserved and rural populations. Given this, it represents a potential gap in the literature and whether we are able to reduce hospitalizations in these underserved communities by embracing these new technologies. 

An important note is that racial information was not directly noted in the various tables of the articles, we can still infer the racial makeup of the patient populations in some studies. For example, in “Effectiveness of a primary care-based integrated mHealth intervention for stroke management in rural China (SINEMA): A cluster-randomized controlled trial”, it is not outright stated that the subjects are of Asian descent, the subjects here were accepted from the Hebei province in Northern China [19]. This occurs in several instances, such as articles from Liberia and Guatemala’s indigenous Maya population [8,18]. 

It is also worthwhile to mention how different countries make the results difficult to compare. Various countries can have vastly different systems of healthcare, resulting in varying results. The SINEMA study provides an excellent look at how healthcare within rural China is provided, where their 3-tier healthcare system can include village doctors who are sponsored by the government as healthcare providers to practice in village clinics and not board-certified physicians [19]. When compared to a system similar to that in Liberia, where clinics are managed by non-governmental organizations (NGOs) as opposed to the government this causes some level of disruption within the analysis. This extends even to within the United States, where the various states responded differently to COVID-19, resulting in widely disparate health outcomes and death rates per 100,000 people [31,32]. However, despite these difficulties, it is still possible to demonstrate the benefits of these technologies in medically underserved communities, despite these differences in culture and geography.

Implications

In summary, we do see benefits when utilizing these technologies; inpatient admission rates in patients who utilize these technologies (i.e., experimental group patients) were hospitalized less frequently compared to patients who did not use these technologies, representing a decrease in overall inpatient hospitalization rates in patients who were able to access care utilizing mobile digital technologies. As per-day hospital costs in the United States, on average, can be $2,883, compared to outpatient doctors’ visits costing an average of $397 for an uninsured patient [33,34]. Costs could also be a limiting factor for rural patients, both in terms of access to various mHealth technologies and in inpatient hospitalization that may result from the underutilization of these technologies.

However, this area does not appear to be as well-researched as it might be in less underserved areas. Given the potential cost benefits these technologies could provide and how they could improve care in areas with fewer healthcare providers, it may benefit both public health and private practice to evaluate whether utilizing these remote monitoring technologies could help providers make better biomedical decisions, such as by providing patient vital signs or improving the frequency of patient concerns, despite the vast distances between patient and provider.

## Conclusions

A mHealth technology has emerged as a promising tool to bridge healthcare gaps in recent years, with these technologies becoming more readily available since the SARS-CoV-2 pandemic. Prior research uncovered in this scoping review provides evidence that these technologies have the potential to optimize healthcare delivery and reduce hospitalization rates in medically underserved areas. However, despite this evidence, there remains a lack of literature compared to other healthcare populations. While several explanations for this could exist, such as a lack of patient education in these areas and insufficient funding, further research may provide additional insight into the effect these devices have on reducing patient hospitalizations. Addressing these questions will not only advance our understanding of how mHealth technologies can improve patient outcomes in underserved areas but also guide policy decisions aimed at improving health equity by expanding access to these crucial tools. Through targeted research, we can ensure that mHealth technologies are deployed effectively to reduce disparities and improve the overall health of underserved populations.
